# Prevalence of, risk factors for, and oxidative stress associated with Toxoplasma gondii antibodies among asymptomatic blood donors in Egypt

**DOI:** 10.1186/1742-4690-9-S1-P27

**Published:** 2012-05-25

**Authors:** Manar S Azab, Nashwa K Abousamra, Mohammad H Rahbar, Doaa M Elghannam, Douaa Raafat

**Affiliations:** 1 Departments of Parasitology, and Clinical Pathology, Faculty of Medicine, Mansoura University, Mansoura 35516, Egypt Divisions of Epidemiology and Biostatist, Egypt

## Background

Since existing therapies are not fully effective, and no Toxoplasma gondii vaccine is available, efforts to reduce toxoplasmosis transmission are crucial to reducing the impact of this disease.

## Objectives

To evaluate the seroprevalence of, risk factors for, and oxidative stress associated with T.gondii antibodies in asymptomatic blood donors in northeastern Egypt in a cross-sectional study.

## Methods

Blood donors were recruited (169 men and 61women) from blood banks, Mansoura University Hospital, Egypt. We interviewed blood donors about sociodemographic characteristics and potential risk factors for T. gondii infection using a structured questionnaire. A venous blood sample was taken to document their T. gondii antibody status using enzyme-linked immunosorbent assay (ELISA). Also, serum level of malondialdehyde (MDA) and activity of glutathione peroxidase (GSH-Px) and tocopherol fractions (α, γ, δ) was assessed.

## Results

Overall, 155 (67.4%) of 230 blood donors were positive for anti-T. gondii IgG antibodies and 24 (10.4%) of them were also positive for anti-T. gondii IgG avidity antibodies, which is high compared to many countries. Univariate logistic regression analysis showed an association between T. gondii seropositivity and area of residence, blood type, older ages, level of education, contact with cats, professional contact with farm animals, agricultural activities, washing hands before meals, eating unwashed vegetables, drinking raw milk, eating luncheon or shawerma. In a multivariate logistic regression analysis, eating luncheon or shawerma showed a strong significant association with T. gondii antibodies. T. gondii-seropositive blood donors had significantly higher MDA level paralleled with significant decrease in the level of GSH-Px and tocopherol fractions compared with T. gondii negative blood donors.

## Conclusion

This study highlights that T. gondii is prevalent among healthy blood donors in northeastern Egypt, and that there is a need to introduce T. gondii screening in the blood donation scheme.

